# Creating solutions for a better response: Global Outbreak Alert and Response Network Regional Partners Meeting for  the Western Pacific, November 2024

**DOI:** 10.5365/wpsar.2024.15.5.1319

**Published:** 2025-11-03

**Authors:** Sharon Salmon, Kanae Takagi, Kieh Christopherson, Kuniko Oka

**Affiliations:** a World Health Organization Regional Office for the Western Pacific, Manila, Philippines.; b UNSW Medicine & Health, School of Population Health, University of New South Wales, Sydney, New South Wales, Australia.; c Japan Institute for Health Security (formerly the National Center for Global Health and Medicine), Tokyo, Japan.; d Indo-Pacific Centre for Health Security, Department of Foreign Affairs and Trade, Canberra, Australia.; e Ministry of Health, Labour and Welfare, Tokyo, Japan.

The first hybrid meeting of the Global Outbreak Alert and Response Network (GOARN) partners from the World Health Organization’s (WHO) Western Pacific Region was held on 20–21 November 2024 in Tokyo, Japan. Hosted by Japan’s National Center for Global Health and Medicine (currently the Japan Institute for Health Security) and the Ministry of Health, Labour and Welfare, the meeting aimed to strengthen regional health security by advancing preparedness and responses to emerging public health threats.

The meeting built on GOARN’s strategic theme that emphasizes creating solutions for better responses. (*1*) The meeting’s objectives were to strengthen GOARN partner collaboration to prevent, prepare for, ensure readiness for and respond to public health emergencies; advance health security in the Region through the implementation of the Asia Pacific Health Security Action Framework (*2*) with the engagement of GOARN; and share opportunities for partners to increase their participation in the Network.

Altogether, 45 GOARN partners attended the meeting from 16 countries across four WHO regions (**Table 1**). Representatives from GOARN partner organizations moderated 2 days of technical sessions. The agenda is in **Supplementary Table 1**. Opening remarks by a representative from Japan’s Ministry of Health, Labour and Welfare emphasized Japan's commitment to strengthening regional health security and acknowledged GOARN’s long-standing role in outbreak responses, especially in the Western Pacific, home to approximately one quarter of GOARN’s 320 partners.



**Table 1:** List of participating institutions by country

Country	Institutions and organizations
**Australia**	**Australasian College for Infection Prevention and Control**
	**Australian National University**
	**Global Health Division, Indo-Pacific Centre for Health Security, Department ** **of Foreign Affairs and Trade**
	**National Centre for Immunization Research and Surveillance**
	**National Critical Care and Trauma Response Centre**
	**Queensland Infection Prevention and Control Unit, Queensland Department of Health**
	**Sydney Infectious Diseases Institute and Westmead Hospital**
	**The Asia Pacific Consortium of Veterinary Epidemiology**
	**University of Newcastle**
	**University of Sydney**
	**University of Western Australia**
**Cambodia**	**WHO Representative Office for Cambodia**
**China**	**Chinese Center for Disease Control and Prevention**
**Fiji**	**The Pacific Community, Suva Regional Office**
**Germany**	**Centre for International Health Protection, Robert Koch Institute**
**India**	**Empower School of Health, Public Health Department**
**Japan**	**Association of Medical Doctors of Asia**
	**Graduate School of Medicine, Department of Virology, Tohoku University**
	**Graduate School of Medicine, Osaka Metropolitan University**
	**Hokkaido University**
	**Japan International Cooperation Agency**
	**Japanese Red Cross Wakayama Medical Center**
	**Mie National Hospital**
	**Ministry of Foreign Affairs**
	**Ministry of Health, Labour and Welfare**
	**Nagasaki University**
	**Nara Medical University**
	**National Center for Global Health and Medicine**
	**National Institute of Infectious Diseases**
	**Saga University Hospital**
	**Saint Mary’s Hospital**
	**Toshima Hospital**
**Mongolia**	**WHO Representative Office for Mongolia**
**Papua New Guinea**	**National Department of Health, Ministry of Health**
**Philippines**	**WHO Regional Office for the Western Pacific**
**Republic of Korea**	**Korea Disease Control and Prevention Agency**
**Singapore**	**Centre for Infectious Disease Emergency Response (CIDER), Yong Loo Lin School ** **of Medicine, National University of Singapore**
	**National Centre for Infectious Diseases and Department of Infectious Diseases, ** **Tan Tock Seng Hospital**
**Switzerland**	**WHO headquarters**
**United Kingdom of Great Britain and Northern Ireland**	**International Severe Acute Respiratory and Emerging Infection Consortium**
	**MRC (Medical Research Council) Centre for Global Infectious Disease Analysis, ** **Imperial College London**
**United States of America**	**Bureau for Global Health, US Agency for International Development**
	**East Asia and Pacific Regional Office, and Division of Emergency Operations, Centers for Disease Control and Prevention**
	**University of Nebraska Medical Center**

The first session focused on the broader ecosystem that supports health security through the Asia Pacific Health Security Action Framework (*2*) and the development of interoperable national health emergency workforces aligned with the vision of the Global Health Emergency Corps. (*3*) The GOARN Handbook (*4*) was presented, illustrating the expansion of GOARN’s role beyond outbreak response to include preparedness and readiness.

GOARN’s role in strengthening the health emergency workforce was highlighted through discussion of its Capacity Strengthening and Training Programme, (*5*) which includes a revised outbreak response simulation exercise and an updated online training platform. A representative from the Public Health Operations in Emergencies for National Strengthening in the Indo-Pacific programme (known as PHOENIX), (*6*) funded by the Australian Government, described how their increased partner engagement has boosted participation in capacity-strengthening activities.

In 2024, the Chinese Center for Disease Control and Prevention, Japan’s National Center for Global Health and Medicine and Germany’s Robert Koch Institute jointly delivered GOARN’s Orientation to International Outbreak Response, demonstrating GOARN partners’ commitment to cross-border capacity-strengthening. A representative from the Robert Koch Institute also discussed its involvement in the GOARN Fellowship Programme. Discussions highlighted the need to enhance engagement through strategic groups, (*7*) strengthen cross-regional collaboration and address fellowship access for Pacific island countries.

GOARN partners shared information about deployment experiences, including providing support to Mongolia during a measles outbreak and the Japan International Cooperation Agency’s surge response to a cholera outbreak in Zambia.

Partners presented research on factors influencing the activation of rapid response teams in Papua New Guinea, with findings that could be used to inform strategies to improve future deployments. A separate partner collaboration emphasized the importance of gender-inclusive leadership by conducting research on women’s leadership during health emergencies. Participants also discussed regional research priorities, including workforce development, rapid response team operations and community engagement.

Panellists discussed the urgency of building local resilience and cross-sector coordination to ensure effective emergency responses, especially in Pacific island countries where climate change has heightened community vulnerabilities. Key strategies that were considered included strengthening community preparedness, integrating health and disaster risk management, and enhancing collaboration among governments, nongovernmental organizations and local stakeholders.

In conclusion, GOARN’s vital role in advancing regional health security was reaffirmed. Partners called for regular communication from GOARN and among partners, shared leadership and regional strategic groups to ensure that activities are tailored to the local context and that they engage underrepresented partners. Key priorities included expanding the GOARN Capacity Strengthening and Training Programme (*5*) and addressing deployment barriers, such as institutional, financial and administrative challenges. Mentorship and remote support during deployments were identified as effective ways to boost partners’ involvement in outbreak responses. Partners also committed to advancing operational research and establishing a regional community of practice to enhance response methods and guide future strategies. Overall, the meeting confirmed GOARN partners’ shared commitment to building a more resilient, collaborative and effective regional health emergency response system.
