# Supplementary material for: Creating solutions for a better response: Global Outbreak Alert and Response Network Regional Partners Meeting for the Western Pacific, November 2024
Source: Western Pac Surveill Response J. 2025 Nov 3;15(5 Spec edition):1–4. doi: 10.5365/wpsar.2024.15.5.1319 (PMC12928874; doi:10.5365/wpsar.2024.15.5.1319)
Supplement: Supplementary file 1 [file wpsar-15-1319-s001.pdf]

Supplementary Table 1. **Agenda**

Day 1: Wednesday, 20 November 2024	
**Time**	**Topic**
8:30–9:00	**Registration**
**Opening session**<br>Moderator: Dr Yukimasa Matsuzawa, Deputy Director, Global Outbreak Intelligence, Capacity Building and Deployment Coordination Center, National Center for Global Health and Medicine, Japan<br>9:00–9:30	
	**Welcome**<br>- Dr Manabu Sumi, Director-General, Department of Infectious Disease Prevention and Control, Ministry of Health, Labour and Welfare, Japan<br>- Dr Gina Samaan, Regional Emergency Director, WHO Regional Office for the Western Pacific<br>- Dr Norihiro Kokudo, President, National Centre for Global Health and Medicine, Japan
	**Meeting objectives and agenda**<br>*Meeting Chairs:*<br>- Dr Mugen Ujiie, Director, Global Outbreak Intelligence, Capacity Building and Deployment Coordination Centre, National Centre for Global Health and Medicine, Japan<br>- Dr Sharon Salmon, Technical Officer GOARN, WHO Regional Office for the Western Pacific
**Session 1: The enabled ecosystem**<br>Moderator: Dr Norio Ohmagari, Director, Disease Control and Prevention Centre, National Centre for Global Health and Medicine, Japan<br>9:30–10:30	
9:30–9:40	**Policy background of the formation of Japan Institute for Health Security (JIHS) and its anticipated roles in health systems strengthening (HSS)**<br>*Dr Norihiro Kokudo, President, National Centre for Global Health and Medicine, Japan*
9:40–9:55	**Asia Pacific Health Security Action Framework**<br>*Dr Gina Samaan, Regional Emergency Director, WHO Regional Office for the Western Pacific*
9:55–10:05	**Global Health Emergency Corps**<br>*Mr Christophe Schmachtel, Partnerships Officer, Global Health Emergency Corps (GHEC) secretariat, WHO Headquarters*
10:05–10:15	**Community-centered: GOARN strategy 2022–2026 implementation**<br>*Dr Gail Carson, Chair, GOARN Steering Committee*
10:15–10:25	**GOARN's role in preparedness, operational readiness, and response**<br>*Mr Armand Bejtullahu, Manager, GOARN Operational Support Team, WHO Headquarters*
10:25–10:30	**Discussion**
10:30–11:00	**Health break**
**Session 2: Health emergency workforce capacity strengthening**<br>Moderator: Ms Renee Christensen, WHO Headquarters<br>11:00–12:30	
11:00–11:20	**GOARN capacity strengthening and training**<br>*Ms Renee Christensen, Lead, Capacity Strenthening and Training, GOARN Operational Support Team, WHO Headquarters*
11:20–11:35	**Outbreak response scenario training**<br>*Professor Paul Effler, Partner lead, GOARN Capacity Strengthening and Training*
11:35–11:55	**Public Health Operations in Emergencies for National Strengthening in the Indo-Pacific (PHOENIX)**<br>*Dr Maya Cherian, A/Director of Public Health, National Critical Care Trauma Response Centre, Australia*
11:55–12:30	**Discussion**
12:30–13:30	**Lunch break**

**Session 3: Coordinated partner collaborations**<br>Moderator: Professor Paul Effler, University of Western Australia<br>13:30–15:00	
13:30–13:40	**Cross-regional collaborations**<br>*Dr Zhen Xu, Deputy Director of Center for Global Health, Chinese Center for Disease Control and Prevention (virtual)*
13:40–13:50	**Partnership building through GOARN activities in Japan**<br>*Dr Yukimasa Matsuzawa, Deputy Director, Global Outbreak Intelligence, Capacity Building and Development Coordination Center, National Center for Global Health and Medicine, Japan*
13:50–14:00 pm	**GOARN Fellowship Program**<br>*Dr Andreas Jansen, Head, Federal Information Centre for International Health Protection, Robert Koch Institue*
14:00–14:10	**WHO Collaborating Centre - GOARN**<br>*Dr Basel Karo, Co-Director, WHO Collaborating Centre for GOARN, Information Centre for International Health Protection, Robert Koch Institute*
14:10–15:00	**Discussion**
**Session 4: Networking and cultural event**<br>Facilitator: Ms Renee Christensen, Dr Sharon Salmon and Ms Kanae Takagi<br>15:00–17:00	
15:00–15:10	**Introducing HIVE – GOARN partners in the WHO Western Pacific Region**
15:10–15:30	**Japanese refreshments**
15:30–16:15	**Japanese cultural performance**
16:15–16:45	**Networking**
16:45–17:00	**Close day 1**

Day 2: Thursday, 21 November 2024	
**Time**	**Topic**
9:00–9:10	**Day 2 agenda**
**Session 5: Surge capacities**<br>Moderator: Mr Sean Starmer, Assistant Secretary, Indo-Pacific Centre for Health Security, Global Health Division, Department of Foreign Affairs and Trade Australia<br>9:10–10:30	
9:10–9:50	**Measles in Mongolia**<br>*Dr Socorro Escalante, WHO Representative to Mongolia (virtual)*<br>*Dr Sapna Sadarangani, Senior Consultant - National Centre for Infectious Diseases, Singapore*<br>*Mrs Belinda Henderson, Chief Nurse - Queensland Infection Prevention and Control Unit, Australia*
9:50–10:10	**Collaborative development: Cholera in Zambia**<br>*Dr Kenichiro Kobayashi, Deputy Director, Department of Infectious Diseases, Japanese Red Cross Wakayama Medical Center (virtual)*<br>*Mr Kenichi Ito, Deputy Director General, and Group Director for Health Group 1, Human Development Department, Japan International Cooperation Agency (JICA)*
10:10–10:30	**Discussion**
10:30–11:00	**Health break**
**Session 6: GOARN research: evidence to action**<br>Moderator: Dr Peta-Anne Zimmerman, Board Director, Australasian College of Infection Prevention and Control (ACIPC)<br>11:00–12:30	
11:00–11:30	**Enabling rapid response teams in Papua New Guinea**<br>*Dr Tambri Housen, Infectious Disease Epidemiologist, School of Medicine and Public Health, University of Newcastle, Australia*<br>*Mr Emmanual Hapolo, National Deparment of Health, Papua New Guinea*
11:30–12:00	**Women leaders in health emergencies**<br>*Dr Jocelyn Herstein, University of Nebraska, USA*<br>*Professor Sharon Medcalf, University of Nebraska, USA (virtual)*<br>*Ms Julia Bae, University of Nebraska, USA (virtual)*
12:00–12:30	**Discussion**
12:30–13:30	**Lunch break**
**Session 7: Creating solutions for better response**<br>Moderator: Professor Paul Effler, University of Western Australia<br>13:30–15:00	
13:30–14:00	**Capacity-building and international collaboration for better response in Japan**<br>*Dr Norio Ohmagari, Director, Disease Control and Prevention Centre, National Centre for Global Health and Medicine, Japan*
14:00–14:50	**Panel Discussion - Disaster**<br>*Moderator: Dr Gina Samaan*<br>*Panelists: Mr Sean Starmer, Australian Government Department of Foreign Affairs and Trade (DFAT); Ms Amy Simpson, The Pacific Community; Dr Norio Ohmagari, National Center for Global Health and Medicine (NCGM); Mr Mark Frank, Centers for Disease Control and Prevention (USCDC); Mr Armand Bejtullahu, GOARN*
15:00–15:30	**Health break**

**Session 8: The way forward**<br>Moderator: Dr Gail Carson, Chair - GOARN Steering Committee<br>15:30–16:30	
15:30–16:15	**Discussion: Regional priorities**<br>*Moderation by:*<br>*Dr Sharon Salmon, Technical Officer GOARN, WHO Regional Office for the Western Pacific*<br>*Dr Gail Carson, Chair - GOARN Steering Committee*
16:15–16:30	**Way forward**<br>*Dr Mugen Ujiie, Director, Global Outbreak Intelligence, Capacity Building and Development Coordination Centre*<br>*Dr Sharon Salmon, Technical Officer GOARN, WHO Regional Office for the Western Pacific*
**Closing session**<br>Moderator: Dr Yukimasa Matsuzawa, Deputy Director, Global Outbreak Intelligence, Capacity Building and Deployment Coordination Center, National Center for Global Health and Medicine, Japan<br>16:30–17:00	
	Dr Norio Ohmagari, Director, Disease Control and Prevention Centre, National Centre for Global Health and Medicine, Japan<br>*Dr Gina Samaan, Regional Emergency Director, WHO Regional Office for the Western Pacific*
